# Regurgitation in healthy and non healthy infants

**DOI:** 10.1186/1824-7288-35-39

**Published:** 2009-12-09

**Authors:** Flavia Indrio, Giuseppe Riezzo, Francesco Raimondi, Luciano Cavallo, Ruggiero Francavilla

**Affiliations:** 1 Department of Pediatrics, University of Bari Policlinico Piazza G.Cesare, 70124 Bari, Italy; 2 Laboratory of Experimental Pathophysiology, National Institute for Digestive Diseases, I.R.C.C.S. "Saverio de Bellis" Via Turi, 14, 70013 Castellana Grotte (Bari), Italy; 3 Department of Pediatrics, University Federico II Policlinico Via S Pansini, 12, 80100 Naples, Italy

## Abstract

Uncomplicate regurgitation in otherwise healthy infants is not a disease. It consists of milk flow from mouth during or after feeding. Common causes include overfeeding, air swallowed during feeding, crying or coughing; physical exam is normal and weight gain is adequate. History and physical exam are diagnostic, and conservative therapy is recommended. Pathologic gastroesophageal reflux or gastroesophageal reflux disease refers to infants with regurgitation and vomiting associated with poor weight gain, respiratory symptoms, esophagitis. Reflux episodes occur most often during transient relaxations of the lower esophageal sphincter unaccompanied by swallowing, which permit gastric content to flow into the esophagus. A minor proportion of reflux episodes occurs when the lower esophageal sphincter fails to increase pressure during a sudden increase in intraabdominal pressure or when lower esophageal sphincter resting pressure is chronically reduced. Alterations in several protective mechanisms allow physiologic reflux to become gastroesophageal reflux disease; diagnostic approach is both clinical and instrumental: radiological series are useful to exclude anatomic abnormalities; pH-testing evaluates the quantity, frequency and duration of the acid reflux episodes; endoscopy and biopsy are performed in the case of esophagitis. Therapy with H2 receptor antagonists and proton pump inhibitors are suggested.

## Background

Regurgitation is defined as the passage of refluxed gastric content into the oral pharynx whilst vomiting is defined as expulsion of the refluxed gastric content from the mouth. The frequency of regurgitation may vary largely in relation to age and younger infants up to first month of age are more frequently affected by regurgitation. Gastroesophageal reflux (GER) is the backward flow of stomach contents up into the esophagus or the mouth. It happens to everyone. In babies, a small amount of GER is normal and almost always goes away by the time a child is 18 months old. The consensus statements that comprise the definition of gastroesophageal reflux disease (GERD) in the pediatric population were developed through a rigorous process [1]. Consensus items of particular note were: (i) GERD is present when reflux of gastric contents causes troublesome symptoms and/or complications, but this definition is complicated by unreliable reporting of symptoms in children under the age of approximately 8 years; (ii) histology has limited use in establishing or excluding a diagnosis of GERD; its primary role is to exclude other conditions; (iii) Barrett's esophagus should be defined as esophageal metaplasia that is intestinal metaplasia positive or negative; and (iv) extraesophageal conditions may be associated with GERD, but for most of these conditions causality remains to be established. The prevalence and natural history of gastroesophageal reflux in infants have been poorly documented. In a recent pediatric prospective survey, the 12% of Italian infants satisfied the Rome II criteria for infant regurgitation. Eighty-eight percent of the infants who had completed two-years follow-up period had improved at the age of 12 months. Only one apart 210 infants turned out to have GERD [2].

Diagnostic investigation of infants who regurgitate, but gain weight satisfactorily and do not exhibit other signs or symptoms is not indicated in clinical practice. The North American Society for Pediatric Gastroenterology, Hepatology and Nutrition (NASPGHAN) [3] recommends that, once other causes of vomiting have been ruled out, infants presenting regurgitation and irritability should undergo a two-week therapeutic test involving a hypoallergenic diet and acid suppression, either sequentially or simultaneously. If no improvement is seen, examinations (pH measurement or endoscopy with biopsy) would be indicated after this period [4]. The non-erosive or exclusively histological reflux esophagitis responds well to treatment based on conservative measures and histamine-2 receptor antagonists (H2RAs), of which the most often used in pediatrics is ranitidine [5].

## Clinical Approach

In children is important distinguishing between normal, physiologic reflux and pathological one. Most infants with physiologic regurgitation are happy and healthy even if they frequently spit up or vomit, and babies usually outgrow GER by their first birthday. These patients have no underlying predisposing factors or conditions, growth and development are normal, and pharmacologic treatment is typically not necessary. Patients with pathologic gastroesophageal reflux or GERD frequently experience complications noted above, requiring careful evaluation and treatment.

Symptoms and signs associated with GER are non-specific. Regurgitation, irritability, and vomiting are common both in infants with physiologic GER or GERD [6] and in infant with other diseases such as food allergy [7], persistent crying [8] and so on. Cough and anorexia/feeding refusal were more common in children 1 to 5 years of age than in older children [9]. Several attempts have been made to introduce specific questionnaire in order to evaluate the role of single gastrointestinal symptoms or cluster of symptoms, calculating the discriminative power of the symptom score in patients and controls. In a recent study the items of a validated questionnaire were tested against the pH esophageal 24-h study in children with suspected GERD. Regurgitation/vomiting yielded the best symptom discrimination, and was reported by 46% with abnormal versus 24% with normal pH-study results. A weighted score including the five best discriminating symptoms was positive in 75% versus 44% [10]. Comparing children with abnormal pH studies and healthy controls, a correct diagnosis based on five symptoms could be obtained in 75% and 94%, respectively. Overall, questionnaires are poorly predictive for the severity of gastroesophageal reflux disease, as they do not correlate with esophageal acid exposure as measured by pH-metry and with esophagitis as evaluated by histology of esophageal biopsies [10,11]. The role of the history and physical examination of a child suspected to have GER(D) is to exclude other disorders that present with the same gastrointestinal symptoms and to identify complications of GERD [12].

## Diagnostic Instrumental Approach

As reported above, a diagnostic approach for the "happy spitter" infants, gaining weight satisfactorily and without other signs or symptoms, is not needed. However, diagnostic instrumental tests can be performed in infants frequently present complications, in which it is not easy to identify individuals who truly have GERD. The list of current available diagnostic tools in the management of GERD are reported in table 1.

**Table 1:** List of current available diagnostic tools in the management of GERD

Exam	Advantages	Disadvantage
*24 h Esophageal pH monitoring*	Gold standard for acid reflux Reference data available Reproducibility Portability	The probe is often disconfortable Non acid or gas reflux are not detected

*Esophageal manometry*	Identification of the GER mechanisms Evaluation of the esophageal and sphincter motility pattern Measurement of esophageal length Portability	Limited availability Trained personnel

*Endoscopy*	Description of esophageal mucosal damage Biopsy allows histological description	Anesthesia is needed Trained personnel

*Rx series*	Fine definition of anatomy	Poor information on the GER mechanism Possible aspiration Rx exposure Not portable

*Scintigraphy*	Study of gastric emptying	Radiation exposure Not portable

### Esophageal pH monitoring

Ambulatory 24-h esophageal pH monitoring is currently the best available test for quantifying esophageal acid exposure, particularly in patients presenting with atypical symptoms. Esophageal pH recording provides quantitative data on both esophageal acid exposure and on the correlation between patient symptoms and reflux events. Esophageal acid exposure is defined by the percentage of the 24-h recording time that the pH is < 4.0. Values > 3.5% are considered abnormal. However, pH monitoring is of limited use in preterm infants whose gastric pH is >4 for the 90% of the time making it almost impossible to detect GER by this technique [13,14]. Wireless pH monitoring has superior sensitivity to catheter studies for detecting pathological esophageal acid exposure because of the extended period of recording (48 hours) and has also shown superior recording accuracy compared with catheter equipment. The American Gastroenterological Association (AGA) reported that ambulatory impedance-pH, catheter pH, or wireless pH monitoring (proton pump inhibitor (PPI) therapy withheld for 7 days) is useful to evaluate patients with a suspected esophageal GERD syndrome who have not responded to an empirical trial of PPI therapy, have normal findings on endoscopy, and have no major abnormality on manometry [15].

Although important contributions have been made to assess the diagnostic value of the long-term pH monitoring in any age pediatric groups, only few reports in children have attempted to correlate the pH pattern of reflux with the clinical severity of gastro-oesophageal reflux disease and to determine the ability of the test to differentiate normal subjects from patients with various degrees of reflux disease [16,17]. The reproducibility of the intraluminal oesophageal pH test to discriminate patients with various degrees of reflux disease have produced contradictory results [18,19]. The 24 hour intraesophageal pH monitoring may present false negative results that limit overall sensitivity of the test. Several scoring systems for pH monitoring studies have been developed [20,21] but any system is clearly better than reflux index (RI) [22].

Development of esophagitis was associated with increased acid exposure of the esophagus. The number of reflux episodes lasting more than five minutes was the most significant variable that differentiated patients with esophagitis from those with simple gastro-esophageal reflux disease. The five minute value is currently regarded as the most accurate variable in predicting the occurrence of esophagitis because it reflects the mechanisms of esophageal acid clearing. However, symptoms may not correlate with acid exposure or the presence of esophagitis. This may be because symptoms may result from nonacidic as well as acidic refluxate [23]. A surprising finding relates to the fact that reflux during sleep was not implicated in the occurrence of esophagitis. It is commonly assumed that reflux occurring during sleep can be more dangerous to the esophagus than the awake acid exposure as acid clearing is usually impaired during sleep [24]. Reports on adults have produced strong evidence that nighttime heartburn and GER represent a distinct clinical entity which deserves specific attention in the diagnosis and optimal treatment of GERD [25]. The discriminating power of the pH test is optimal for long lasting recording, even the postprandial esophageal integrated acidity provides a robust estimation of esophageal acid exposure and may predict symptoms in gastro-esophageal reflux disease patients [26]. However, in infants milk or formula feeding can neutralize gastric acidity, so reflux of non-acid gastric content might not be detected by pH test [23].

### Multiple intraluminal esophageal impedance

Gastroesophageal reflux can be acid, nonacid, pure liquid, or a mixture of gas and liquid. Esophageal pH and impedance were used to identify acid reflux (pH drop below 4.0), minor acid reflux (pH drop above 4.0), nonacid reflux (pH drop less than 1 unit + liquid reflux in impedance), and gas reflux [27]. Non-acid reflux is a particular problem in pediatrics because children are fed more frequently than adults and the majority of non-acid reflux occurs in the post-prandial period when stomach content is neutralized.

Additionally, there are many children that are continuously fed through gastrostomy tubes such that the pH of the stomach is neutral for the majority of the day. Other factors can explain a negative pH monitoring in subjects with gastro-esophageal reflux disease. First, episodes of alkaline gastroesophageal reflux might be overlooked using the standard routine pH measurement. Increased flow/volume of saliva can reduce the exposure acid time of the esophagus neutralising the acidity of the refluxed content [28]. Esophageal bile reflux seems to play an additional role in the pathophysiology of gastroesophageal reflux disease [29]. A third possible explanation for a negative pH result in patients with gastro-esophageal reflux disease lies in the variability of the prolonged intraesophageal pH monitoring. In fact, milk fed infants had been reported to have a low reflux index reflecting prolonged buffering of gastric acidity rather than the absence of reflux [30,31].

Previous pediatric studies have shown that between 30-88% of reflux in children is non-acid [32]. The literature has focused on the role that acid reflux plays and currently, it is thought that non-acid reflux may be involved in the pathogenesis of respiratory diseases [33]. Children under the age of 18 months have the highest rates of acute respiratory diseases of any age group. Many of these acute illnesses progress to chronic respiratory diseases such as asthma, which result in significant morbidity and mortality [34]. Despite excellent medical therapy, the prevalence rates of chronic respiratory disease remain high.

A recent work have attempted to characterize the proportion of acid and nonacid esophageal reflux events in young infants with suspected GER using combined pH-multichannel intraluminal impedance (pH-MII) monitoring. To determine the symptom index correlation with nonacid reflux and acid reflux events in children, aged 2 weeks to 1 year, 1890 reflux events were detected by pH-MII, and 588 reflux events were detected by pH probe alone. The percent of reflux that was acid was 47% versus 53% of nonacid reflux events. The proportion of nonacid reflux decreased with age and with increasing time elapsed from last meal. The most frequently reported symptom was fussiness/pain, which correlated with nonacid reflux events 24.6% and acid reflux 25.2%. The proportion of nonacid reflux to acid reflux events in infants was more similar to adults than previously reported. Combined pH-MII esophageal monitoring identifies more reflux events and improves clinical correlation with symptoms [35].

The pH-MII catheter is a small tube that is inserted through the nose into the esophagus and is identical in size to the standard pH probe. The catheter remains in place for 24 hours during which it continuously measures the amount of both acid and non-acid reflux that is entering the esophagus from the stomach. Another significant advantage to pH-MII is the ability of the catheter to measure the height of the refluxed stomach contents; impedance sensors are positioned throughout the esophagus so reflux extends along the entire length of the esophagus, and even up into the mouth and potentially the airway, can be determined. Pediatric studies have suggested that the pH-MII catheter is as sensitive as the pH probe in the detection of reflux.

This tool has been very useful in the evaluation of patients with atypical reflux symptoms (such as asthma, chronic cough, laryngitis, chest pain) and in patients who continue to have symptoms while taking acid blocking medicines. Studies in adults and children have shown that the addition of pH-MII monitoring significantly improves the physicians' ability to diagnose reflux-related disease. In studies of infants, the use of pH-MII has been particularly important in clarifying the relationship between respiratory diseases. While the association between apnea and reflux in infants has been debated, there is some evidence that non-acid reflux may be associated with breathing problems in these young patients. In a study of infants with primarily respiratory symptoms who underwent pH-MII testing, the standard pH probe failed to detect 88% of reflux episodes that were associated with breathing problems [36]. There is also literature that suggests that non-acid reflux in children, as well in adults, may be associated with other respiratory symptoms. In particular, in children with severe respiratory disease who were taking acid blocking medicine, non-acid reflux seems more likely to be associated with respiratory symptoms than acid reflux. In pediatrics, pH-MII has been used to evaluate other reflux therapies such as body positioning [37], apnea of premature infants [38], and thickening of feeds [39]. All of the therapeutic studies have involved a small number of patients and additional data on the treatment of non-acid reflux are needed.

Because the understanding of the role of non-acid reflux is in its infancy, very few studies have addressed the treatment options for patients with pathologic non-acid reflux. Adult and pediatric studies suggest that proton pump inhibitors such as omeprazole and lansoprazole do not decrease the total amount of reflux in patients. Instead, they convert the reflux from acid to non-acid reflux which may explain why some patients continue to have symptoms despite therapy with proton pump inhibitors [40]. Adult studies have suggested that therapy with the drug baclofen may effectively treat non-acid reflux [41]. Baclofen is a gamma-aminobutyric acid (GABA) agonist which decreases the amount of esophageal sphincter (LES) relaxations, the main cause of reflux. However, because of its evident side effects on the central nervous system (CNS) (drowsiness, confusion or mental depression, mood or mental changes, seizures) baclofen is undesirable for use as a treatment for GERD. Further development work has yielded a number of novel GABA type B receptor agonists with reduced CNS side effect profiles, and clinical trials are currently being performed with several agents. Compounds that target esophageal sphincter relaxations may therefore present a new add-on treatment for patients with persistent GERD symptoms despite PPI therapy.

### Upper endoscopy and histology

Endoscopy associated with histology is a reliable and accurate method to demonstrate esophageal damage induced by GERD, such as inflammation and strictures. However, up today optimization and standardization of pediatric endoscopy procedure have not yet realized [42]. The findings of erythema, edema, loss of shine and friability in the distal esophagus are aspecific, and the introducing of controversial parameters for esophagitis diagnosis, with interpretations varying greatly from one endoscopist to another, have increased the disagreement between macroscopic and histological findings. In contrast, the presence of esophageal erosion is less subject to observer interpretation [43]. Some authors observed that enanthema of the esophageal mucosa may not have any histological correspondence with reflux esophagitis. Studies have shown the predominance of disagreement between endoscopic and histological results in milder cases, while agreement between the two diagnostic tests predominates in more severe forms [44]. Hiatal hernia is the only endoscopic observation that predicts erosive esophagitis [45]. The use of the Tytgat classification, which does not take into account the presence or absence of Barrett's esophagus, but describes non-erosive abnormalities observed in the discrete esophagitis (commonly observed among infants), may report the endoscopic diagnosis of level I esophagitis associated with normal histology [46].

All patients with erosive esophagitis presented reflux esophagitis on histology. The esophageal biopsy plays an important role, as much in cases of normal examinations or mild abnormalities as in cases of erosive esophagitis. If the edema, erythema and friability commonly observed in children are non-specific, findings from histological examination and morphometric studies of the esophageal mucosa allow an etiologic diagnosis of eosinophilic esophagitis if characteristic alterations such as eosinophil infiltrates, increased total epithelial and basal cell thickness, and elongation of stromal papillae are seen [47]. Furthermore, histopathology allows the investigation of other diagnostic possibilities such as infectious esophagitis (Herpes virus, Cytomegalovirus, Candida), Barrett's esophagus, dysplasia, adenocarcinoma, Crohn disease, and others. Microscopic evaluation of biopsy samples from the distal esophagus, but avoiding the most distal area to minimize the false positive findings at LES, demonstrated abnormalities in many patients who have symptoms but no endoscopically evident erosions. Infiltration of the epithelium with inflammatory cells, the changes recognizable in esophageal epithelium regardless of orientation of the specimen, received early attention. Neuthrophils and eosinophils are not normally present in the ephitelium of the children and can be used as marker of GERD even though they may be fairly insensitive [48]. Intraepitelial limphocites are more sensitive than other inflammatory cells but they are very common and so their specificity for GERD remains unclear.

Eosinophilic oesophagitis results in inflammation the esophagus, and in most cases are seen in people with allergies such as hay fever and asthma. There is some evidence that this may be an unusual form of food allergy. It is important to rule out it since eosinophilic esophagitis can progress to esophageal stenosis, and not responding well to anti-GER treatment, corticoid therapy being indicated instead. In such cases, the high eosinophil density (> 20 per high power field) and the presence of eosinophils in the proximal esophagus favor the hypothesis of eosinophilic esophagitis [49]. To determine the clinical, endoscopic, and histologic criteria that distinguish children with eosinophilic esophagitis (EE) from those with non-EE diagnoses, a retrospective case-control study was performed for children with any degree of esophageal eosinophilic inflammation who underwent esophageal biopsy [50]. Although EE and non-EE patients complained of vomiting and abdominal pain at equivalent rates, EE patients were 3 times more likely to complain of dysphagia and twice as likely to have stricture formation. On endoscopy, patients with EE were 19-times more likely than non-EE patients to have endoscopic abnormalities. Histologically, EE patients were more likely to have basal zone hyperplasia and degranulated eosinophils [50]. Although the above mentioned findings, the histologic distinction between EE and GERD cannot be reliably made on histopathologic evidence alone in children with upper aerodigestive symptoms. Despite the recent gastroenterology consensus statement regarding the clinic-pathologic diagnosis of EE, children with primary airway symptoms in whom EE is suspected represent a diagnostic dilemma [51].

### Motility studies

Motility disorders are postulated to potentially cause reflux since an association between diminished LES tone, transient LES relaxations, delayed gastric emptying and GER have been recognized. Esophageal manometry measures movement and pressure in the esophagus. In particular, it measures esophageal motility pattern and coordinated peristalsis, and the upper and lower esophageal sphincter pressures. There are two main types of manometric recording systems: perfused and solid state. Both have strengths and weaknesses, and the choice of any particular system depends on how these strengths and weaknesses are viewed. Esophageal motility develops during infancy and early childhood, and may be influenced by various factors, including maturation, dietary and postural habits, arousal state, ongoing illnesses, congenital anomalies, and effects of medical or surgical interventions. Esophageal motility is particularly important because it regulates the movement of a bolus during swallowing or during GER. Infantile reflux is different from adult reflux in that regurgitation or vomiting is quite common, even in normal infants [52]. Despite its common occurrence, the mechanisms of esophageal and airway protection during episodes of GER in infants are relatively poorly understood. None of the current approaches [3] for the evaluation of GER in infants evaluates the protective mechanisms. To date, there is not much evidence of esophageal defense mechanisms against GER in children, although data exist from adult studies [53,54]. In summary, carefully performed esophageal manometric studies in infants and children should include (1) basal measurements of the esophageal body and sphincters; (2) details of post-prandial state, including the response to wet and dry swallows; (3) response to esophageal provocation; and (4) identification of esophageal-protective reflexes. Such information may be useful in understanding the pathophysiology of esophageal motor function [55].

Manometric study is useful in identifying transient relaxations of the LES as a pathophysiological mechanism of GERD [56] and for the diagnosis of achalasia or other motor disorders of the esophagus which may present itself as reflux. Esophageal motor abnormalities are commonly found in children with esophagitis [57] and in children with developmental delay and neurologic impairment, with GERD recurred after Nissen funduplication [58]. As regard the discriminating role of manometric studies, a recent study point out that manometry assess only resting LES pressure and its length in children with acid GER but do not clearly differentiate GER into primary and secondary refluxes to cow's milk allergy [59]. Gastric emptying studies have shown prolonged half-emptying times in children with gastroesophageal reflux. The significance of this phenomenon is not clear. Tests of gastric emptying are not routinely performed in patients with suspected GERD, but may become worthy gastric retention is suspected (see scintigraphy and ultrasonography).

### Imaging

#### Radiography

Plain radiographic findings are not useful in evaluating patients for GERD, but they are helpful in evaluating pulmonary status and basic anatomy. Esophageal inflammatory and neoplastic diseases are better detected with double-contrast techniques [60]. Conversely, single-contrast techniques are more sensitive for structural defects such as hiatal hernias and strictures or esophageal rings [61]. Various techniques are used, and each has relative strengths and weaknesses in the ability to detect specific abnormalities or disease processes. A typical barium esophagram is performed in multiple steps or phases. A high-density barium suspension is administered, and double-contrast views are used for images taken with the patient in the upright position. Prone-positioned images are typically obtained with single contrast and a lower-density barium suspension. Mucosal relief images can be made to complement these techniques.

Early esophagitis is not well demonstrated and decreases the overall sensitivity of barium swallows [62]. This is why many clinicians reserve barium swallow for the evaluation of patients with GERD and symptoms that include dysphagia. Barium swallow is not sensitive in the detection of actual reflux, except in the occasional patient who has a wide-open LES and free reflux. Radiographic series are neither sensitive nor specific for diagnosing GERD especially compared to tests such as 24-hour pH monitoring. The presence of Barrett esophagus occasionally is detected as a reticular mucosal pattern. As expected, the more advanced the esophageal disease, the more sensitive is barium swallow at detecting it [63]. Barium swallow is a very important study in the investigation and detection of postoperative complications following fundoplication. Recurrent hiatal hernia, disruption or slippage of the fundoplication, and other structural abnormalities can be identified [64]. Late postoperative dysphagia can be investigated by a combination of manometry and esophageal fluoroscopic examination. Increases in esophago-gastric transit time of liquid barium and solid boluses correlate positively with the presence of postoperative dysphagia [65].

#### Ultrasonography

Conventional ultrasonography have reported to be a reliable non invasive method to detect reflux events and as well to describe anatomical conditions such as hiatal hernia, length and position of the LES and the magnitude of the gastro-esophageal angle of His. Although conventional sonography is not a diagnostic tool for achalasia, it provides interesting sonographic information. It cannot reveal each layer of the wall of the lumen as endoscopic ultrasound does, but it may tentatively differentiate achalasia from malignancies and assists clinicians when endoscopic ultrasound is not available [66]. Few improvements have been introduced for studying esophageal function, i.e high-frequency intraluminal ultrasound, whereas conventional techniques, such as manometry, have undergone substantial upgrades because of advances in transducer technology, computerization, and graphic data presentation. Although this techniques provide both novel and more detailed information regarding the measure of the esophageal contractility and the thickness of esophageal muscle, it is still unclear whether they have improved the ability to diagnose and treat patients more effectively [67,68]. Ultrasonography is not recommended as a test for GERD for its low sensitivity and specificity.

Last, dinamic ultrasound may be useful for the study of the gastric emptying time [69,70]. Antral measurements are made before and immediately after the end of the test meal (time 0), and at regular 30-min intervals up to 180 min after the meal. In each patient, the gastric emptying rate was expressed as percent reduction in antral cross sectional area from time 0 to 120 min after meal ingestion [71]. Gastric emptying assessed by a non-invasive technique as ultrasonography is particularly suitable for young patients even if it is time consuming and investigator dependent [72].

In young children suspected of GERD, the gastroesophageal junction was examined with ultrasonography directly after a feeding while these children were on overnight extended esophageal pH monitoring (EEpHM). The two tests showed 81% to 84% agreement in the detection of the presence or absence of GER, depending on whether the whole period of EEpHM or only the part of it covering the ultrasound observation period [73]. The two studies probably measure different aspects of clinically significant reflux and must be correlated with the clinical symptoms. Morphological findings associated with significant reflux were: (1) a short intra-abdominal part of the esophagus, (2) a rounded gastroesophageal angle, and (3) a "beak" at the gastroesophageal junction. Barium meal findings confirmed these sonographic signs, indicating a sliding hiatal hernia of the distal esophagus, either fixed or intermittent. Ultrasonography can be recommended as a useful and physiological screening test to demonstrate clinically significant GER and a predisposing hiatal hernia of the esophagus in symptomatic children but it is not routinarily used in the diagnosis of GERD.

#### Scintigraphy

Gastroesophageal reflux and clearance of the refluxed material can be measured by plotting a time-activity curve from an esophageal area of interest after 1 mCi of^99m^Tc sulfur colloid is placed in the stomach. Control subjects do not have peaks exceeding a value twice that of the baseline count levels. Reflux patients exceed this value, either spontaneously or after Valsalva maneuvers. This technique has a sensitivity which is greater than that of barium and equal to the sensitivity of a pH probe in patients with both moderate and severe reflux. Scintigraphic reflux was shown in 62% of moderate refluxes and 85% of those with severe reflux as defined clinically. This test can be performed rapidly with minimal radiation exposure and is noninvasive [74]. The sensitivity of the milk scan compared to pH probe for diagnosis of esophageal reflux is 15-59% that is low whilst specificity is much higher since it is 83-100% [75]. Scintigraphy in children with GERD can provide information on postprandial reflux and delayed gastric emptying [76]. Besides, the 1-hr scintigraphic study formatted in 60-sec frames provides a quantitative representation of postprandial gastroesophageal reflux for children, particularly if they do not have rapid gastric emptying [77]. Even its ability to identify reflux and gastric emptying time, the routine diagnosis and management of GERD in infants and children does not comprise scintigraphy.

## Treatment

The treatment of GER/GERD should be individually tailored according to the clinical manifestation and possible complications. Treatment options for regurgitation and GERD include conservative measures, dietary management, pharmacologic therapy and surgery. Table 2 contains the strategy step and the grade of recommendation for each of them [78].

**Table 2:** Therapeutic options in gastro-oesophageal reflux in neonates, infants and children according with the strategy steps and the grade of recommendation

Therapeutic option	Strategy Step	Grade of recommendation
Positioning	1	GRADE B (the left lateral position)

Feed frequency	1	GRADE D

Thickened formula or feed	2	GRADE B (for reducing vomiting)

Domperidone	3	GRADE C/D

Ranitidine/cimetidine + PPI	3	GRADE B/C (in relieving esophagitis)

Surgery	4	Surgical intervention is rarely necessary in case of severe complications

### Conservative measures

Because most cases are functional GER, reassurance is the only treatment needed [79]. Conservative measures may include upright positioning after feeding, elevating the head of the bed, prone positioning (infants >6 mo), and providing small, frequent feeds thickened with cereal [80]. Older children benefit from a diet that avoids tomato and citrus products, fruit juices, peppermint, chocolate, and caffeine-containing beverages. Smaller, more frequent feeds are recommended, as well a relatively lower fat diet because lipid retards gastric emptying [81]. Prone positioning may be recommended, at least for the first postprandial hour [82]. Clearly, the use of the prone position during infancy must be based on a careful risk-to-benefit analysis. When it is advised, only very firm bedding material (no pillows) must be used. Bed elevations offer no added advantage to the prone position, and seated positions are not recommended.

### Dietary management

Although some authors consider conservative therapy to be an efficient first choice for improving regurgitation even compared with thickened formula [79], the latter has been considered a reliable dietary management for decreasing recurrent regurgitation and/or vomiting in young infants [83]. Several thickening agents, i.e. rice cereal, gelatin, carob bean gum or galactomannan, have been successfully administered for the treatment of regurgitation in infants [84,85] and they provide a therapeutic advantage, particularly when excessive vomiting is associated with suboptimal weight gain [86]. Even for infants with normal weight gain, thickened and reduced volume feedings may reduce the frequency and amount of vomiting episodes, ameliorating the concerns of an anxious caregiver. Formula thickened with carob flour, locust bean gum, rice cereal or rice starch have been found to decrease episodes of regurgitation and vomiting as well as esophageal acid exposure [83,87]. Undesiderable side effects may occur, however, with various thickening agents. Orestein et al. reported an increase in coughing after infants were fed a formula enriched with rice cereal [88] and Takahashi et al. reported that soybean fibre decreased food consumption and weight gain in an animal model [89]. Clarke and Robinson [90] reported some cases of fatal necrotising enterocolitis in infants fed carob thickened milk.

As a result, the last European Society for Pediatric Gastroenterology Hepatology and Nutrition (ESPGHAN) guidelines suggested avoidance of formula thickened with locust bean in infants up to six months because the possible risk of enterocolitis [91]. Thus, there is a need for alternative interventions to thickening agents in infants with recurrent regurgitation. Probiotic formulas have been shown to promote a regression of symptoms [71] without adverse growth or behavioral effects whilst the earlier demonstration of the safety and tolerance of probiotics in full term infants makes it suitable for use in this population [92]. Further understanding and elucidation of the mechanisms underlying the beneficial effects of probiotics on gastrointestinal symptoms and motility should provide new regimens for prevention and treatment of illness in infants. Another possible diet option could be feeding the infant with a formula supplemented with prebiotics. Prebiotics stimulate the gastric emptying so improving tolerance to enteral feeding, and this would be of clinical relevance. However, this hypothesis needs further evaluation [93].

The leading symptoms of GERD are present in the case of cow milk allergy. This disorder should be considered in preterm infants with recurrent vomiting and irritability [94]. Confirmation of this diagnosis and treatment consists of a trial of cow milk protein free formula. In some cases infants are also allergic to hydrolysate and so the only treatment is amino-acid based formula [13].

### Pharmacologic therapy

In the case of pharmacologic intervention, "step-up" therapy involves progression from diet and lifestyle changes to H2RAs and to PPI [3]. Both classes of acid antisecretory have proven safe and effective for both infants and children in reducing gastric acid output [95]. A specific target may be children with moderate-to-severe neurodevelopmental disabilities who typically have manifest dysphagia and gastroesophageal reflux, and present a high risk for aspiration [96,97]. In these patients, conservative therapy alone may not be sufficient in preventing reflux-associated complications. Overall, the therapeutic approach of GERD disease in infants and children needs to be well-balanced, considering therapeutic efficacy and side effects of the different therapeutic options [98]. Last, careful monitoring under optimal nonsurgical therapy should be conducted before considering operative intervention [96,99].

H2RAs decrease acid secretion by inhibiting H2 receptors on gastric parietal cells [100]. The fairly rapid tachyphylaxis that develops with H2RAs is a drawback to chronic use. In some infants, H2RA therapy causes irritability, head banging, headache, somnolence and other side effects which, if interpreted as persistent symptoms of GER, could result in an inappropriate increase in dosage [101]. H2RAs, particularly ranitidine, are associated with an increased risk of liver disease, and cimetidine with gynecomastia [102].

PPIs inhibit acid secretion by blocking Na+, K+ ATP-ase, the final common pathway of parietal cell acid secretion, often called the proton pump. PPIs currently approved for use in children in North America are omeprazole, lansoprazole and esomeprazole. In Europe, only omeprazole is approved. No PPI has been approved for use in infants < 1 year of age. Most studies of PPIs in children have demonstrated the efficacy of PPIs in the controlling of symptoms and haeling of erosive esofagitis [103,104]. Children 1-10 yrs of age appear to have a greater metabolic capacity for some PPIs than adolescents and adults; that is, they require higher per kilogram doses to attain the same acid blocking effect, or area-under-the-curve [105]. There are few pharmacokinetic data for PPIs in infants, i.e. lansoprazole displays pharmacokinetic and pharmacodynamic parameters in children between 13 and 24 months of age similar to those observed in older children and adults [106]. Infants < 6 months may have a lower per kilogram dose requirement than older children and adolescents. In preterm infants and term neonates esomeprazole produces no change in bolus reflux characteristics despite significant acid suppression [107]. Last, a recent study detected no difference in efficacy between lansoprazole and placebo for symptoms attributed to GERD in infants age 1 to 12 months. Severe adverse events, particularly lower respiratory tract infections, occurred more frequently with lansoprazole than with placebo [108].

### Surgery

When medical therapy has failed, or when complications of gastroesophageal reflux are present [109], the antireflux operations may include partial or complete fundoplication and, if possible, the reduction of the hiatal hernia [110]. As pharmacotherapy has improved, the need for surgical therapy has markedly decreased. Nevertheless, antireflux surgery remains one of the most common surgical procedures performed during infancy and early childhood for refractory erosive oesophagitis or reflux aspiration [111]. Current guidelines from NASPGHAN [3] have reported the conditions in which surgery may be suggested. GERD with an atypical presentation, especially respiratory, whose symptoms are clearly associated with gastroesophageal reflux (i.e. obstructive apnea temporally associated with reflux during pH monitoring) should be considered for surgical treatment. However, a period of medical therapy (including acid blockade) under close monitoring conditions should be attempted in many cases prior to recommending a surgical approach. Besides, patients with complications of gastroesophageal reflux, such as aspiration, stricture of the esophagus, or Barrett esophagus should be considered for surgical treatment. In particular, children with pathologic reflux and neurologic impairment, that requires feeding gastrostomy and continuous medication should also be considered for surgery. For those infants who fail medical therapy, continuous intragastric administration of feeds via nasogastric tube is an option [112]. It is often used in preterm infants because of the significantly greater surgical risk in such patients. In these cases, adequate nutritional management, in conjunction with appropriate medical therapy, may permit the infant to "outgrow" reflux while optimizing weight gain.

There are no controlled studies of fundoplication versus medical therapy and studies evaluating different surgical treatments. In fact, there is no randomization of children undergoing partial versus complete wraps, even if some studies suggest that the results of partial one was better than those of Nissen fundoplication [112]; there are no clinical trials comparing laparoscopic antireflux surgery versus open antireflux ones. Only retrospective reviews and case series have been performed demonstrating laparoscopic antireflux procedures safe and effective once the learning curve has been achieved [113]. Complications of fundoplication include dysphagia for solid food, gas bloat syndrome, wrap herniation and dumping syndrome.

## Conclusion

Standard approaches to infants who regurgitate gastric contents (often the overflow from an overly generous feeding) differ from that recommended for children who reflux and have resultant disease manifestations (GERD). For infants with functional GER, a rational and conservative approach is to reassure the parents of the benign nature of the "spitting". Pathologic gastroesophageal reflux or gastroesophageal reflux disease refers to infants with regurgitation and vomiting associated with poor weight gain, respiratory symptoms, esophagitis. In such case clinical and instrumental diagnosis are needed. Among the latter upper radiology, pH-testing and MII testing are useful for diagnosis. Endoscopy and biopsy are performed in the case of esophagitis. The therapy with H2 receptor antagonist is currently suggested.

## List of Abbreviations

AGA: American Gastroenterology Association; EE: Eosinophilic esophagitis; ESPGHAN: European Society for Pediatric Gastroenterology Hepatology and Nutrition; EEpHM: Extended esophageal pH monitoring; H2RA: Histamine2 receptor antagonist; LES: Lower esophageal sphincter; GABA: Gamma-aminobutyric acid; GER: Gastroesophageal reflux; GERD: Gastroesophageal reflux disease; NASPGHAN: North American Society for Pediatric Gastroenterology Hepatology and Nutrition; pH-MII: pH-Multichannel intraluminal impedance; PPI: Proton pump inhibitor; RI: Reflux index.

## Competing interests

The authors declare that they have no competing interests.

## Authors' contributions

FI: Conceived the study and helped draft the manuscript. GR: Drafted the manuscript. FR: Participated in the drafting and polishing the manuscript in the diagnostic approach section. LC: Participated in its design and coordination RF: Participated in its design and coordination. All authors read and approved the final manuscript.
